# Light induces oxidative damage and protein stability in the fungal photoreceptor Vivid

**DOI:** 10.1371/journal.pone.0201028

**Published:** 2018-07-20

**Authors:** Carmen Noemí Hernández-Candia, Sergio Casas-Flores, Braulio Gutiérrez-Medina

**Affiliations:** 1 Division of Molecular Biology, Instituto Potosino de Investigación Científica y Tecnológica, San Luis Potosí, Mexico; 2 Division of Advanced Materials, Instituto Potosino de Investigación Científica y Tecnológica, San Luis Potosí, Mexico; Consejo Superior de Investigaciones Cientificas, SPAIN

## Abstract

Flavin-binding photoreceptor proteins sense blue-light (BL) in diverse organisms and have become core elements in recent optogenetic applications. The light-oxygen-voltage (LOV) protein Vivid (VVD) from the filamentous fungus *Neurospora crassa* is a classic BL photoreceptor, characterized by effecting a photocycle based on light-driven formation and subsequent spontaneous decay of a flavin-cysteinyl adduct. Here we report that VVD presents alternative outcomes to light exposure that result in protein self-oxidation and, unexpectedly, rise of stability through kinetic control. Using optical absorbance and mass spectrometry we show that purified VVD develops amorphous aggregates with the presence of oxidized residues located at the cofactor binding pocket. Light exposure increases oxidative levels in VVD and specific probe analysis identifies singlet oxygen production by the flavin. These results indicate that VVD acts alternatively as a photosensitizer, inducing self-oxidative damage and subsequent aggregation. Surprisingly, BL illumination has an additional, opposite effect in VVD. We show that light-induced adduct formation establishes a stable state, delaying protein aggregation until photoadduct decay occurs. In accordance, repeated BL illumination suppresses VVD aggregation altogether. Furthermore, photoadduct formation confers VVD stability against chemical denaturation. Analysis of the aggregation kinetics and testing of stabilizers against aggregation reveal that aggregation in VVD proceeds through light-dependent kinetic control and dimer formation. These results uncover the aggregation pathway of a photosensor, where light induces a remarkable interplay between protein damage and stability.

## Introduction

Flavin-binding photoreceptors mediate blue-light (BL) sensing in diverse organisms [1,2], and have received considerable attention recently as genetically-encoded fluorescent and optogenetic tools [3,4]. Light perception mechanisms in these proteins involve light absorption by the chromophore, followed by rearrangements of electronic structure at and near the flavin, resulting in a conformational change that drives the photoreceptor to its signaling state [5]. A particular class of BL photoreceptors, Light-Oxygen-Voltage (LOV) domain proteins present a characteristic photocycle, where BL induces formation of a cysteinyl-flavin adduct (the lit state) that spontaneously decays back to the ground (dark) state [6]. Despite significant advances in understanding the photochemistry of flavin-based photosensors, the rich chemistry of the flavins [7,8] suggests that there may be multiple paths along which light signals could progress [6].

Typically, light absorption in LOV domains results in singlet excited states that decay to the ground state (through fluorescence) or to excited triplet states (through non-radiative intersystem crossing) that in turn participate in photoadduct formation [9,10]. Alternatively, significant energy transfer from the excited triplet state to molecular oxygen (with the subsequent production singlet oxygen, ^1^O_2_) has been achieved by artificially suppressing adduct formation in the engineered LOV domain mini Singlet Oxygen Generator (miniSOG) [11]. In native flavoproteins, cryptochrome 1 from *Arabidopsis thaliana* has been shown to undergo flavin reoxidation from the fully reduced or the neutral radical states with production of reactive oxygen species (ROS) [12]. However, little is known on the capacity of BL receptors in general to produce ROS, the potential self-oxidizing effects of ROS products, and the functional consequences of such modifications. These processes gain relevance as chemical damage is known to turn a native structural conformation into misfolded [13,14], from where protein aggregation can proceed as intermolecular interactions driven mainly by hydrophobic forces develop [15]. To date only a few cases of aggregation in BL photoreceptors have been explored [16,17].

One of the most studied LOV photoreceptors is the protein VIVID (VVD), a regulator of photoadaptation in the filamentous fungus *Neurospora crassa* [18]. VVD is constituted by a single LOV domain that binds a flavin adenine dinucleotide (FAD) as cofactor, and its mechanism of BL perception is well known. Briefly, the dark state of VVD presents a characteristic optical absorption spectrum with three main peaks (λ = 428, 450 and 478 nm). BL incident on VVD induces the formation of the photoadduct formed between the C4a carbon of FAD and the sulfhydryl group of a highly-conserved cysteine (C108), producing a single absorption peak around 390 nm [18]. Adduct formation triggers a conformational change that releases the N-terminus of VVD and enables its interaction with the transcription factor White Collar-1 (WC-1), inhibiting the transcriptional activity of the White Collar Complex (WCC) formed by the WC-1 and the White Collar-2 (WC-2) proteins [19,20]. Photoadduct spontaneous decay regenerates the dark state and completes the photocycle. Full-length, recombinant VVD has been reported unstable under native conditions [18], but a truncated version of VVD (VVD36) increases stability [19]. Yet, VVD36 stored in oxygenated buffer was found to get oxidized at Cys71 [21] and to aggregate [22]. Nevertheless, the possibilities of VVD to act as a photosensitizer have not been explored and the factors leading to VVD aggregation are not well understood. The role of light in the aggregation of this photoreceptor is of particular interest.

Herein, we report use of aggregation and biochemical assays, electron microscopy, and mass spectrometry to test the effects of light and presence of cosolutes in the aggregation process of VVD. We found that VVD develops amorphous aggregates with oxidation at residues corresponding to the flavin-binding pocket region, suggesting an inside-out production of ROS. Furthermore, by testing the effect of diverse scavenger molecules in aggregation and using specific fluorescent probes we identify singlet oxygen as the damaging reactive species. Our analysis of the aggregation kinetics under dark and light conditions shows that the first step towards aggregation is dimer formation and, surprisingly, that the VVD lit state suppresses protein aggregation. These results uncover the aggregation pathway of VVD, demonstrate that VVD acts not only as a photoreceptor but as a photosensitizer, and show that light has an additional unexpected role as inductor of protein stability.

## Materials and methods

### VVD expression, purification and storage

All reagents were purchased from Sigma-Aldrich unless otherwise specified. Wild-type VVD truncated at the N-terminus by 36 residues and containing an N–terminal 6×His tag (plasmids kindly provided by Dr. Brian R. Crane, Cornell University), were over expressed in *Escherichia coli* BL21(DE3) cells. After reaching an Optical Density (OD) at 600 nm of 0.6 to 0.8 the culture was induced with 100 μM IPTG and left for 22 h under room lighting and constant shaking at 18°C. Harvested cells were flash frozen and stored at -80°C for subsequent protein purification. Thawed cells were resuspended at 4°C in Lysis Buffer (150 mM NaCl, 50 mM HEPES, 10% glycerol (v/v), pH 8) supplemented with 1% Triton X-100 and protease inhibitor cocktail tablets (EDTA free). Protein was purified using a NTA-Nickel (QIAGEN, cat. 30210) affinity resin in a gravity column at 4°C (this step was not performed under strict dark conditions). Purified protein was stored in the dark at –20°C using storage buffer (150 mM NaCl, 50 mM HEPES, 100 mM imidazol, 50% glycerol (v/v) and pH 8). Stored protein remained stable for months at the high glycerol concentration used. Protein concentration was determined using the Bradford assay.

### Transmission electron microscopy sample preparation

Standard procedures were followed for negative staining using gadolinium acetate tetrahydrate. Briefly, an aggregated sample was centrifuged and the supernatant was discarded. The aggregated protein pellet was resuspended and washed with water three times, and finally resuspended in alcohol. A drop of aggregated protein sample was deposited in a carbon formvar grid and left to settle during 15 min. Next, 1 mL of water was used to wash the grid and 5 μL of 10% gadolinium acetate tetrahydrate (Ted Pella, cat. 19485) were added, incubated for 1 min, and then removed with filter paper. Finally, 5 μL of water were added on the sample, incubated for 5 min, and the water excess was removed with filter paper. Completely dry samples were observed in a transmission electron microscope JEOL200CX.

### Release of the flavin cofactor

A VVD sample at 67 μM in standard buffer (SB; 20 mM imidazole, 150 mM NaCl, 50 mM HEPES, pH 8, and 10% (v/v) glycerol) was illuminated for 5 min and then incubated at 25°C. After an incubation time of 24 h the VVD sample became visibly turbid. Centrifuging of the turbid sample (10 min at 13,000 rpm) separated aggregated protein. The supernatant was passed through a centrifugal filter (Millipore Amicon Ultra-15 10K) to retain soluble protein. The retained and through fractions were analyzed by recording their corresponding absorption spectrum.

### Aggregation kinetics

We characterized the kinetics of protein aggregation in VVD using turbidity as a metric, measuring the absorbance at λ = 550 nm (*A*_550_) as a function of time. Stored protein samples were resuspended to the proper glycerol concentration in resuspension buffer (RB, 50 mM HEPES, 150 mM NaCl, pH 8) and VVD concentration was fixed at 31 μM unless otherwise specified. After preparation samples containing 150 μL each were immediately loaded on the flat bottom of a 96–well plate, and 70 μL of mineral oil was added at the top of each well to avoid evaporation. To establish the initial condition, a BL LED (Sink PADII, Royal-Blue, λ = 440–460 nm, 1 W, Luxeon) was used to illuminate the plate for 5 min (at ~1 mW/cm^2^). Illumination using a red LED (Sink PADII, Deep Red, λ = 650–670 nm, 1 W, Luxeon) did not produce any effect in VVD absorption spectra. Next, samples were then taken to a microplate spectrophotometer (Synergy, Biotek), and absorption spectra (300–600 nm) were acquired every 30 min at controlled temperature (25°C). All proteins or molecules used to probe effects on the aggregation of VVD (GSH, DTT, trolox, dimethylurea, propyl gallate, BSA, linoleic acid-oleic acid-albumin, superoxide dismutase, catalase, and glycerol) were first resuspended in SB, added fresh to VVD samples; then, prepared VVD samples were subject to a 5-min BL pulse and absorption spectra recorded every 30 min.

### Protein electrophoresis and Western blot

To confirm the presence of VVD oligomers in turbid samples, an initially-fresh VVD sample was placed at 25°C for 24 h after a 5-min BL pulse. After centrifuging, the pellet was analyzed using standard SDS–PAGE and Western blot assays against VVD´s 6×His-tag using a Penta-His biotin conjugate (QIAGEN, cat. 34440) and an ultra–sensitive Streptavidin–Peroxidase Polymer. To evaluate oxidation levels in VVD samples, carbonyl groups were detected in three different sample conditions at the same protein concentration. A sample illuminated for 5 min with BL, a sample without illumination, and an intentionally oxidized VVD sample (by incubating during 15 min with 1.5 mM NiCl_2_, 33 mM H_2_O_2_ under BL illumination) were supplemented with 5 mM biotin-hydrazide and left to incubate for 3 h at room temperature. Ultra-sensitive streptavidin peroxidase polymer was used to detect the biotin-hydrazide label. To test the effect of DTT on aggregation, fresh VVD samples were subjected to a 5-min BL pulse and then incubated at 25°C for 16 h in the presence of 1 mM or 10 mM DTT. After incubation samples were resuspended in Laemmli buffer with or without 10% 2-β-Mercaptoethanol (BME) and SDS-PAGE was performed.

### Mass spectrometry

The pellet fraction of an aggregated sample was loaded on a SDS-PAGE gel. The protein band was excised from the gel and reduced with 10 mM DTT, 25 mM ammonium bicarbonate, followed by protein alkylation with 55 mM iodacetamide. Protein was digested with Trypsin Gold (PROMEGA, V5280). Next, nanoscale LC separation of tryptic peptides was performed using a nanoACQUITY UPLC System (Waters, Milford, MA, USA) and tandem mass spectrometry analysis was carried out in a SYNAPT HDMS (Waters). MS/MS data sets were used to generate PKL files using the Protein Lynx Global Server v2.4 (PLGS, Waters). Peptides were then identified using PKL files and the MASCOT software. Searches were conducted against the NCBI protein database. Data were analyzed using 20 ppm peptide mass tolerance, 0.6 Da fragment mass tolerance, P = 0.05 significant threshold and one missed cleavage allowed. Carbamidomethyl cysteine was set as fixed modification and oxidation of D, K, P, R, F, M, N, and Y were specified as variable modifications.

### Aggregation kinetics analysis

The starting condition to begin aggregation kinetics measurements was a sample of purified VVD stored in the dark, at -20°C, and under 50% (v/v) glycerol. In this storing condition all proteins remained stable for weeks. As environmental conditions changed to induce aggregation (see below) we followed the kinetics of aggregate formation by recording *A*_550_.

### Dark reaction

A sample of stored VVD protein at 50% (v/v) glycerol was placed at 25°C and left for 6 h in the dark for complete photoadduct decay. Next, the sample was taken to 10% (v/v) glycerol and left to aggregate (25°C) while maintaining the protein in the dark. We model the aggregation kinetics according to a second-order reaction where the irreversible formation of the final aggregated state (VVD-A) depends on the interaction between two dark-VVD proteins. The aggregation reaction follows:

$$\frac{dA}{dt}={k_{1}N}^{2}$$

$$\frac{dN}{dt}=-k_{1}N^{2},$$

Where *A* is the protein concentration in the final aggregated fraction (VVD-A), *N* is concentration of dark-VVD proteins in the soluble fraction, and *k*_1_ is the reaction constant. In this case, protein can be in only two states: either soluble (dark-VVD) or aggregated (VVD-A), that is *A* + *N* = *N*_0_, where *N*_0_ is the initial concentration of soluble VVD. Solving for *A* provided the initial conditions *A*(*t* = 0) = 0 and *N*(*t* = 0) = *N*_0_:

$$A\left(t\right)=N_{0}\frac{N_{0}k_{1}t}{N_{0}k_{1}t+1}.$$

During amorphous aggregation turbidity is linearly proportional to aggregate concentration in the low optical absorbance regime (< 0.5) [23]. Therefore, the fitting function to our data is:

$$A_{550}\left(t\right)=CN_{0}\frac{N_{0}k_{1}t}{N_{0}k_{1}t+1},$$

where *C* is a constant. Fitting to data corresponding to VVD initially prepared in the dark-state with *N*_0_ = 31 ± 6 μM (estimated error from the Bradford assay) yielded *C* = 6.3 ± 1.3×10^3^ M^-1^ and *k*_1_ = 2.3 ± 0.5 M^-1^ s^-1^.

### Light reaction

VVD protein samples were placed at 25°C and 10% (v/v) glycerol, subject to 5-min BL pulse and left to aggregate. Here, light induces adduct formation, placing the protein in the lit-VVD state that we assume unable to aggregate. Upon adduct decay the protein returns to the dark-VVD state and becomes competent to dimerize with another dark-VVD protein. The reaction rates for *A* and *N* (defined above) are now:

$$\frac{dA}{dt}={k_{1}N}^{2}$$

$$\frac{dN}{dt}=-k_{1}N^{2}+k_{2}e^{-k_{2}t},$$

where *k*_1_ is the second-order reaction constant and *k*_2_ = 1/τ with τ being the adduct decay time of VVD. The initial conditions in this case are *A*(*t* = 0) = 0 and *N*(*t* = 0) = 0. The above system of differential equations cannot be solved analytically. Numerical integrations were performed (using Mathematica), keeping the rate parameter *k*_1_ fixed during analysis (at value *k*_1_ = 2.3 M^-1^ s^-1^). A least squares routine was used to find best fits to experimental aggregation data, yielding τ = 3.4 ± 0.1 ×10^3^ s (mean ± SD).

## Results

### VVD displays amorphous aggregation at room temperature

**Fig 1:**
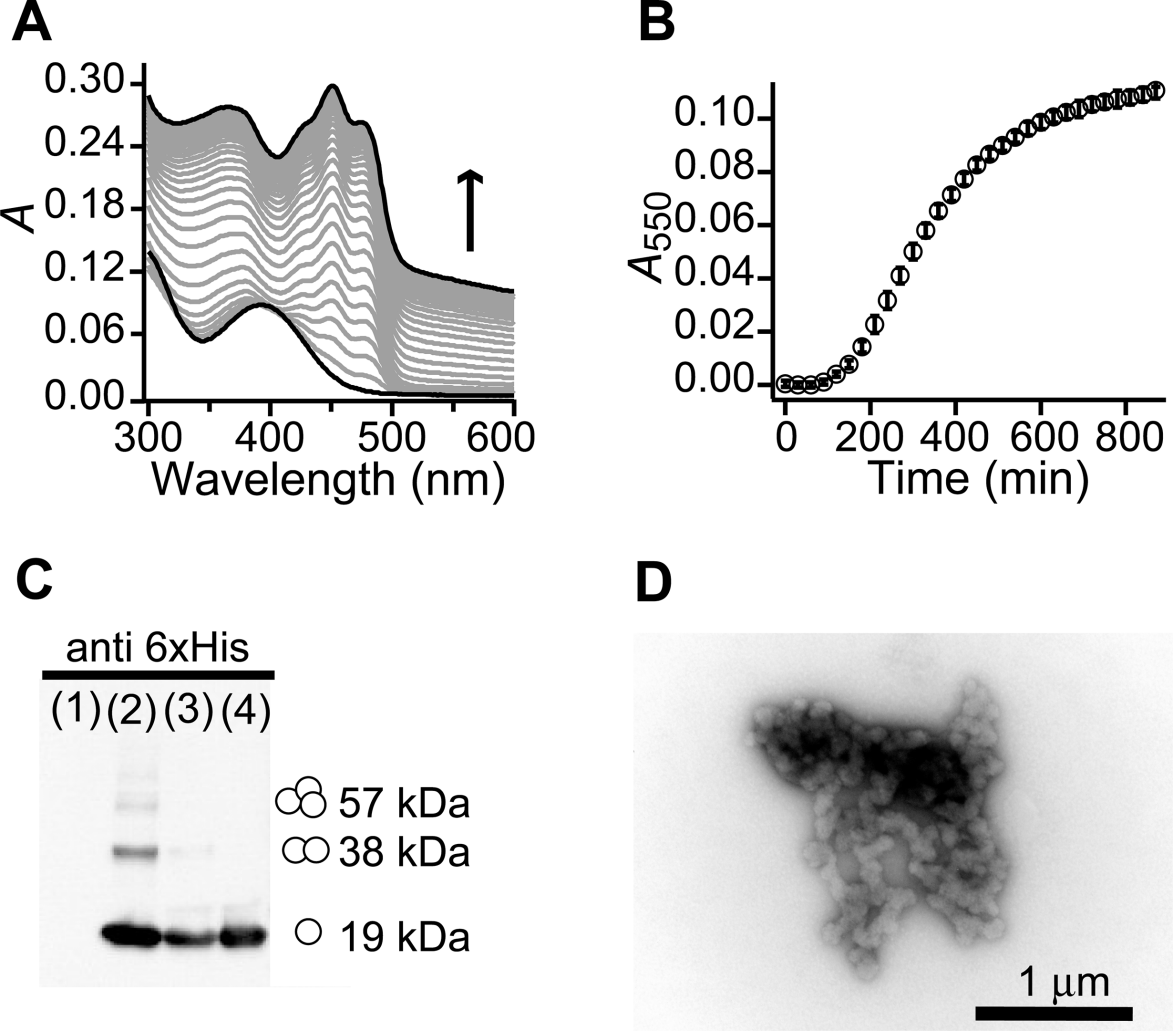
VVD exhibits amorphous aggregation at standard conditions. (A) The absorbance (*A*) spectra of VVD (0.56 mg/mL, 31 μM) at 25°C and standard buffer conditions (10% glycerol, 50 mM HEPES, 150 mM NaCl, 20 mM imidazole, pH 8) acquired every 30 min after a BL pulse, show a shift in absorbance. First and last spectra are shown in black. Arrow indicates time course of the absorbance shift. (B) The aggregation of VVD was quantified by measuring the absorbance at λ = 550 nm. A representative VVD aggregation kinetics record (0.56 mg/mL) is shown (mean ± SD, *N* = 3). (C) A Western blot against VVD´s 6×His shows that samples with an absorbance shift form VVD oligomers. Control samples in lanes 1 and 4 correspond to bovine serum albumin (BSA) and unaggregated protein, respectively. The pellet and the supernatant of a centrifuged aggregated VVD sample were loaded in lane 2 and 3, respectively. The pellet fraction presents three bands at molecular weights corresponding to monomer (19 kDa), dimer (38 kDa) and trimer (57 kDa) of VVD. (D) Transmission electron microscopy of VVD samples with absorbance shifts display amorphous aggregation.

We tested the photoadduct decay kinetics of VVD (VVD36, a version with the first 36 amino acids deleted and a 6×His tag at the N-terminus [19]) by measuring optical absorbance under test buffer conditions (25°C, pH 8, 150 mM NaCl, 50 mM HEPES, 20 mM imidazole, 10% (v/v) glycerol). As full-length recombinant VVD is reported not soluble above 4°C [18], use of stable VVD36 afforded us a functional protein as a starting condition in our experiments. Purified protein kept at -20°C under 50% glycerol remains soluble for months. A protein sample diluted from 50% to 10% glycerol was illuminated with BL (5 min) to induce adduct formation. After illumination the absorption record displayed a main peak at 390 nm and no absorption beyond 500 nm (Fig 1A), indicating that initially all proteins were soluble and well-folded so as to allow effective adduct formation. However, a clearly noticeable absorbance shift systematically presented at all wavelengths (Fig 1A), increasing with time, due to a change in turbidity usually associated with protein aggregation [24]. To quantify the amount of aggregated VVD we measured absorbance at λ = 550 nm (*A*_550_), a wavelength where soluble VVD does not absorb. The aggregation kinetics displays an initial lag phase followed by sustained growth over hundreds of minutes (Fig 1B). A Western blot analysis against VVD´s 6×His-tag showed that the macroscopic pellet recovered after spin down of the turbid sample indeed consisted of monomers, dimers, trimers, and higher molecular oligomers of VVD (Fig 1C). We noticed that VVD’s aggregated pellet exhibited a whitish color instead of the characteristic yellow hue associated to flavoproteins (Figure A in S1 File), and the presence of free FAD in the supernatant (Figure A in S1 File). The protein pellet was further visualized by Transmission Electron Microscopy (TEM), evidencing a mesh of amorphous aggregates (Fig 1D). These results show that VVD develops aggregates formed by proteins that have released the non-covalently bound FAD molecule, implying that some of the protein structure is lost during aggregation.

To avoid protein aggregation common recombinant protein troubleshooting tests were conducted (varying buffer and purification conditions or removing the 6×His tag, see Supporting Information and Figure B in S1 File), none of which presented observable effect. Additionally, we stress that the aggregation of VVD is not caused by conditions such as high temperature, extreme pH or addition of chemical denaturants. These facts suggest that VVD develops aggregates due to factors related to the intrinsic function of the protein within standard conditions.

### VVD shows oxidative damage and produces singlet oxygen

**Fig 2:**
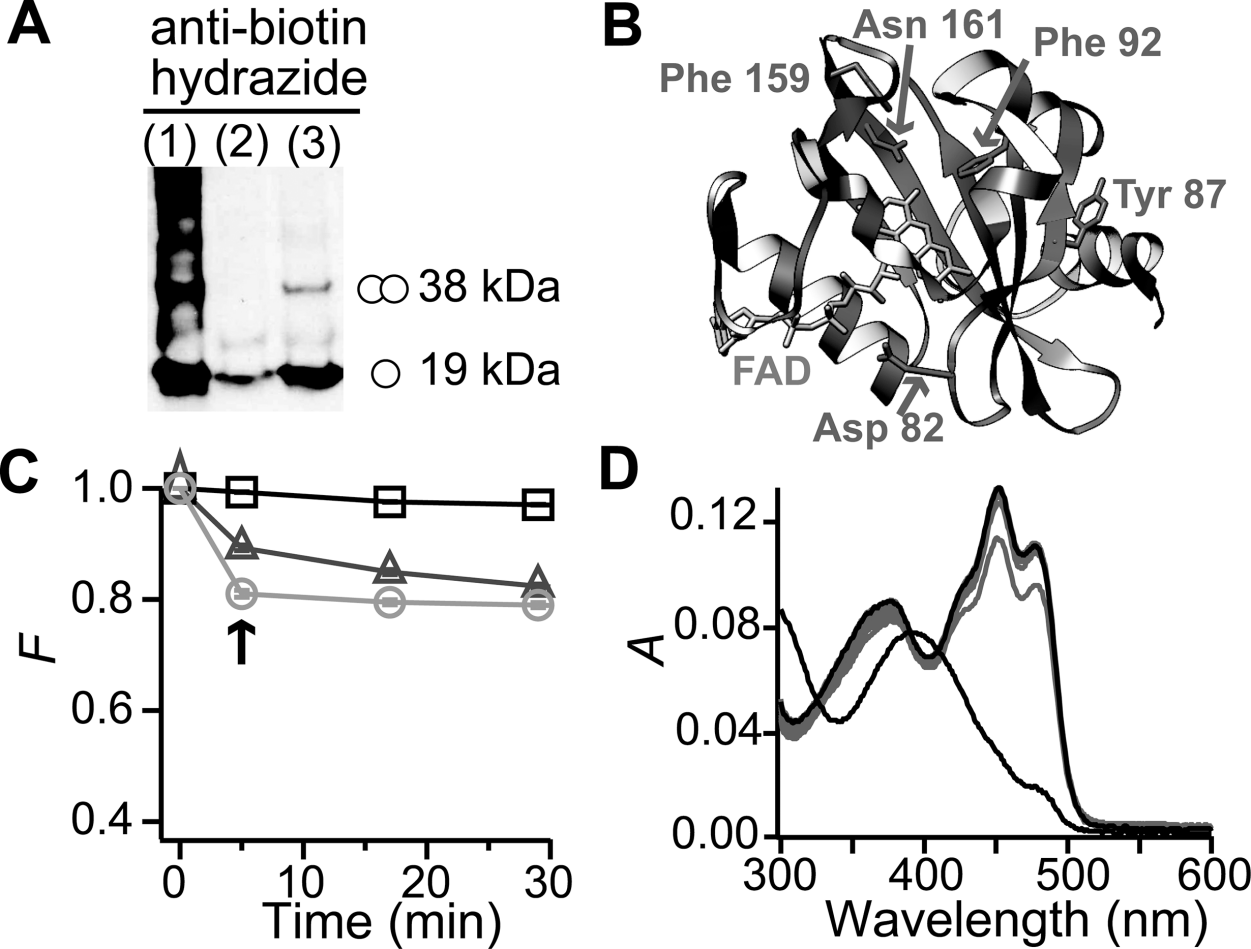
VVD becomes oxidized by self-produced singlet oxygen. (A) Detection of oxidized proteins by Western blot against biotin-hydrazide in labeled samples. Lane1: VVD intentionally oxidized using 33 mM H_2_O_2_ and 1.5 mM NiCl_2_. Lane 2: VVD sample not illuminated. Lane 3: VVD sample illuminated for 5 min with BL. (B) The 3D structure of VVD in the dark state (Protein Data Bank 2PD7) highlighting the oxidized amino acids detected by mass spectrometry of aggregated samples. (C) Fluorescence intensity (*F*) records of AMDA in the absence and presence of free FAD and VVD. At *t* = 0 samples were subject to BL illumination during 5 min. In the presence of VVD (triangles) bleaching of AMDA is observed as a reduction in *F* immediately after illumination (arrow). In control experiments, free FAD (a known producer of ^1^O_2_) causes fluorescence bleaching (circles) and BL has no effect on AMDA alone (squares). Data were normalized with respect to the value of *F* before BL illumination. [VVD] = 30 μM, [FAD] = 30 μM, [AMDA] = 10 μM. Data: mean, error bars: SD; *n* = 3. (D) Addition of *A*. *niger* catalase (2.34 μM) avoided aggregation and shortened adduct mean lifetime to τ ≈ 1.1×10^3^ s.

VVD carries a photosensitizer molecule as cofactor, thus protein oxidation emerged as a possible mechanism to disrupt VVD stability towards aggregation. We evaluated oxidation in VVD samples using a biotin-hydrazide assay reactive to carbonyl groups [25]. Protein subjected to 5 min of BL presented increased carbonyl content with respect to non-illuminated protein (Fig 2A). To pinpoint the oxidized residues, the pellet recovered from a centrifuged aggregated sample was analyzed by tandem mass spectrometry (MS/MS), showing oxidation in amino acids: D82, Y87, F92, F159, and N161 (Fig 2B and Figure C in S1 File). Remarkably, F92 and N161 are located inside the protein and are not exposed to the solvent, whereas F159 is partially exposed to the solvent with its lateral chain pointing towards the internal part of the protein.

Oxidation of internal amino acids suggests that the flavin is acting as a photosensitizer, producing reactive oxygen species (ROS) that chemically modify the protein and disrupt stability. We sought to find the identity of the oxidant molecule involved by testing the effects of diverse ROS scavengers on aggregation. Trolox, mannitol, Cu/Zn-superoxide dismutase, propyl gallate, and dimethylurea did not show any effect on aggregation (Figure D in S1 File). By measuring fluorescence quenching of the specific ^1^O_2_ probe 9,10-Anthracenediyl-bis(methylene)dimalonic acid (AMDA) as it reacted with VVD after a BL illumination pulse we found that VVD is capable of producing ^1^O_2_ (Fig 2C), supporting the role of VVD as a photosensitizer.

Notably, high concentrations of catalase from *Aspergillus niger* (2.3 μM) were found to effectively suppress aggregation in VVD (at 31 μM), with the added effect of shortening VVD´s adduct decay lifetime by well over three-fold (see Fig 2D and below). This unexpected modification of the adduct decay rate and the high concentration of catalase needed to observe the effect suggests that catalase did not act as a conventional hydrogen peroxide scavenger to prevent protein aggregation, implying a reaction route of VVD and its cofactor not identified before.

### The aggregation of VVD is under kinetic control and regulated by photoadduct dynamics

**Fig 3:**
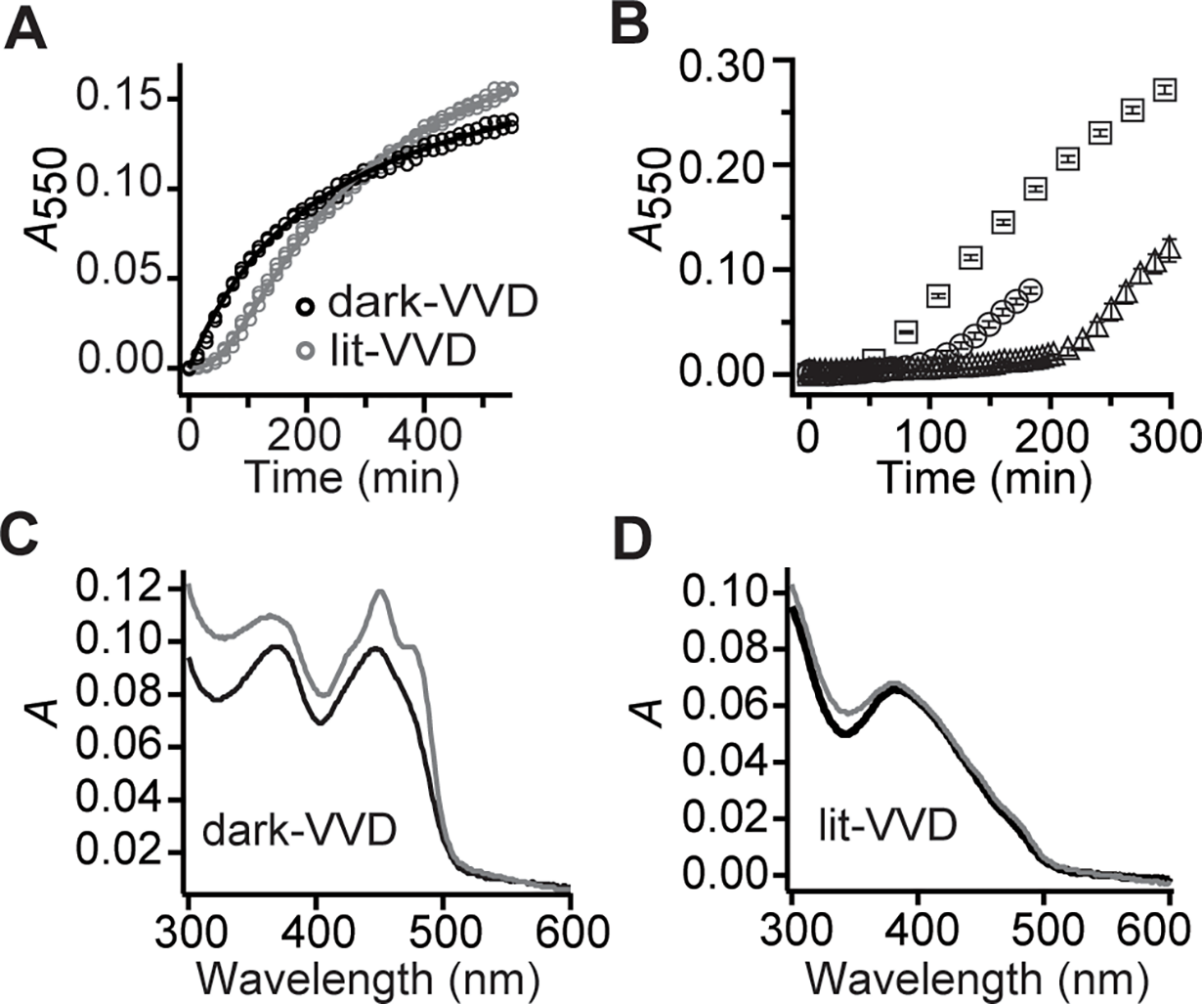
The aggregation of VVD is under kinetic control and regulated by photoadduct dynamics. (A) Aggregation kinetics of VVD initially prepared in a dark-state or lit-state. The kinetics records are well fit (solid lines) by a second-order reaction model that considers photoadduct decay as the limiting step for VVD aggregation. Both samples were diluted in standard buffer (10% glycerol, 50 mM HEPES, 150 mM NaCl, 20 mM imidazole, pH 8). The lit-state corresponds to the same conditions tested in Fig 1B. (B) Aggregation halts during the illumination cycling period and resumes only after samples are returned to the dark. Samples were initially illuminated for 30 s and their aggregation kinetics was followed. 5-s BL pulses were applied every ~5 min, over 68 min (circles) or 202 min (triangles). Aggregation kinetics of a sample subjected to only the initial BL pulse is included for reference (squares). Data: mean, error bars: SD; *n* = 3. (C-D), VVD lit-state is resistant to denaturant conditions whereas dark-state is not. Proteins initially prepared in a dark-state (C) or in a lit-state (D) were challenged with 0.01% SDS (black records) or left in standard buffer without SDS (gray records), and their absorption spectra were immediately acquired.

We investigated whether aggregation proceeds indistinctly from the VVD dark or light states. Protein samples were prepared in each of these states by storing in darkness (dark-VVD) or subjecting to a 5-min BL pulse (lit-VVD), and the aggregation kinetics were followed by measuring *A*_550_ as a function of time (Fig 3A and Figure E in S1 File). We found that the dark-VVD state proceeded to aggregate without delay. In contrast, the lit-VVD state presented aggregation kinetics with an initial lag phase (lasting ~1 h, see also Fig 1B) during which development of aggregation was minimal. These observations suggest that the limiting step for aggregation is the decay of lit-VVD photoadduct. To test this idea, VVD samples were subjected to short (5 s) BL pulses applied every 5 min, with the aim to repopulate the lit state. As this cycling procedure was extended for a maximum of 68 or 202 min, it resulted in suppression of aggregation during these times (Fig 3B and Figure F in S1 File). These results demonstrate that the aggregation of VVD is under kinetic control and that formation of the flavin-cysteine adduct originates stability in VVD.

### The aggregation kinetics in VVD is consistent with light-induced stability and aggregation of dark VVD through dimer formation

From the previous experiments, we observed that: (i) the lit-VVD state is stable and it is only the dark-VVD state (resulting after photoadduct decay) that is competent to aggregate, and (ii) the dark-VVD state can still perform effective photoadduct formation with subsequent protein stability (over a 5-min time interval, Fig 3B). Taking these facts into account, the aggregation kinetics data were modeled according to the following second-order reactions

$$2\;\mathrm{dark}‑\mathrm{VVD}\overset{k_{1}}{\to }\mathrm{VVD}‑A$$ (1)

and

$$\mathrm{lit}‑\mathrm{VVD}\overset{k_{2}}{\to }\mathrm{dark}‑\mathrm{VVD}$$

$$2\;\mathrm{dark}‑\mathrm{VVD}\overset{k_{1}}{\to }\mathrm{VVD}‑A$$ (2)

for samples initially prepared in the dark-VVD and lit-VVD states, respectively, where VVD-A is the amount of aggregated protein, *k*_1_ is the second-order rate constant of the conversion of dark-VVD into aggregate, and *k*_2_ = 1/*τ*, with *τ* being the photoadduct decay time in VVD (see Materials and Methods). Experimental records were well fitted by the models (Fig 3A), yielding *k*_1_ = 2.3 ± 0.5 M^-1^ s^-1^, and *τ* = 3.4 ± 0.1×10^3^ s. The value for *τ* found here is consistent with previous measurements of photoadduct lifetime in VVD36 (22) and our own measurements of protein at 50% (v/v) glycerol, where no aggregation is present (data not shown). From this analysis we conclude that the observed lag phase in aggregation records (Fig 1A) is due to photoadduct decay and that although the dark-VVD state is aggregation-competent it is only through dimer formation that aggregation ensues. The aggregation kinetics of samples at different initial protein concentrations are also well described by the propose model (Figure G in S1 File).

To further explore the stability of the lit-VVD state, we performed a conventional test to identify kinetically-trapped proteins. Resistance against denaturation was evaluated by exposing the lit or dark protein states to 0.01% sodium dodecyl sulfate (SDS), resulting in immediate loss of the flavin cofactor only for the dark state, where the sample exhibits the absorption spectrum of free flavin (Fig 3C). In contrast, the lit state displays its characteristic photoadduct spectrum with and without SDS (Fig 3D), consistent with an increased resistance to denaturation compared to the dark state.

### Specific stabilizing factors limit aggregation in VVD

**Fig 4:**
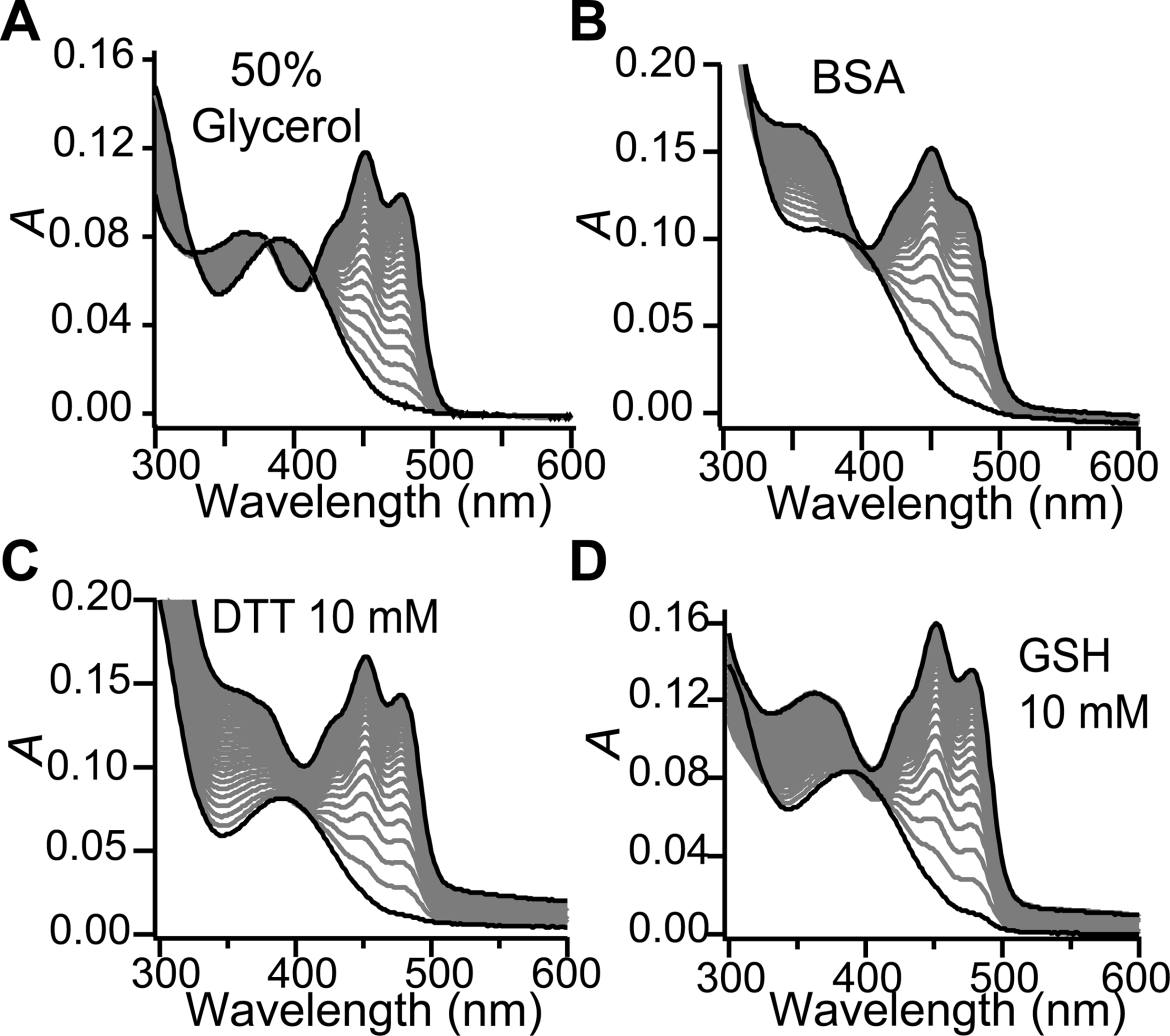
Effect of various stabilizing factors on the aggregation of VVD. (A) Addition of the osmolite glycerol fully avoids protein aggregation, with recovery of the three reported isosbestic points at wavelengths: 330 nm, 385 nm, and 413 nm (18). (B) Addition of BSA (at 242 μM) avoids VVD aggregation. The addition of chemically reducing agents DTT (C) and GSH (D) also limited aggregation. First and last absorption spectra are shown in black; data were acquired every 30 min.

We examined the mechanism of dimer formation in the aggregation of VVD by testing the effect of various stabilizing factors. Glycerol has been reported to drive proteins towards conformationally compact states, possibly by acting as an amphiphilic interface between hydrophobic protein surfaces and the polar solvent [26], thus preventing the destabilizing exposition of hydrophobic regions. Increased concentrations of glycerol in VVD samples resulted in reduction of protein aggregation (Figure H in S1 File). At 50% (v/v) glycerol the aggregation of VVD is fully avoided (Fig 4A) even after several phototcycles.

Bovine serum albumin (BSA) is known to feature a chaperon-like activity by binding to hydrophobic-exposed sites of proteins [27]. We found that BSA also limited protein aggregation in a dose-dependent way (Figure H in S1 File), and at high BSA concentrations (242 μM) the rise of turbidity was nearly stopped (Fig 4B). At such high BSA concentrations, however, crowding effects on VVD cannot be discarded. To discriminate between chaperon and crowding effects, fatty acids known to occupy the hydrophobic regions of BSA and inhibit its chaperon activity [27] were tested, with the result that BSA no longer prevented the aggregation of VVD (Figure H in S1 File). Therefore, the effects of glycerol and BSA suggest that exposure of hydrophobic regions in VVD is involved in dimer formation and aggregation.

We observed that the small molecules Dithiothreitol (DTT) and Glutathione (GSH) succeeded in significantly reducing aggregation (Fig 4C and 4D), pointing to possible involvement of disulfide bonds in dimer formation during the aggregation of VVD. This inference was confirmed by allowing purified protein to aggregate in the presence of DTT. Analysis using SDS-PAGE electrophoresis (Figure I in S1 File) showed limited formation of VVD oligomers when DTT was present. DTT and GSH are also known singlet oxygen scavengers; however, this activity is unlikely here as we observed suppression of VVD aggregation even when GSH or DTT were added 5 min after a BL pulse (data not shown)–a time interval orders of magnitude longer than the expected lifetime of ^1^O_2_. Lastly, a C71S VVD mutant unable to perform the light-triggered conformational change [19] also displayed aggregation (Figure J in S1 File), indicating that the dimer species in aggregation is of different nature form the known light-induced dimer [21].

## Discussion

**Fig 5:**
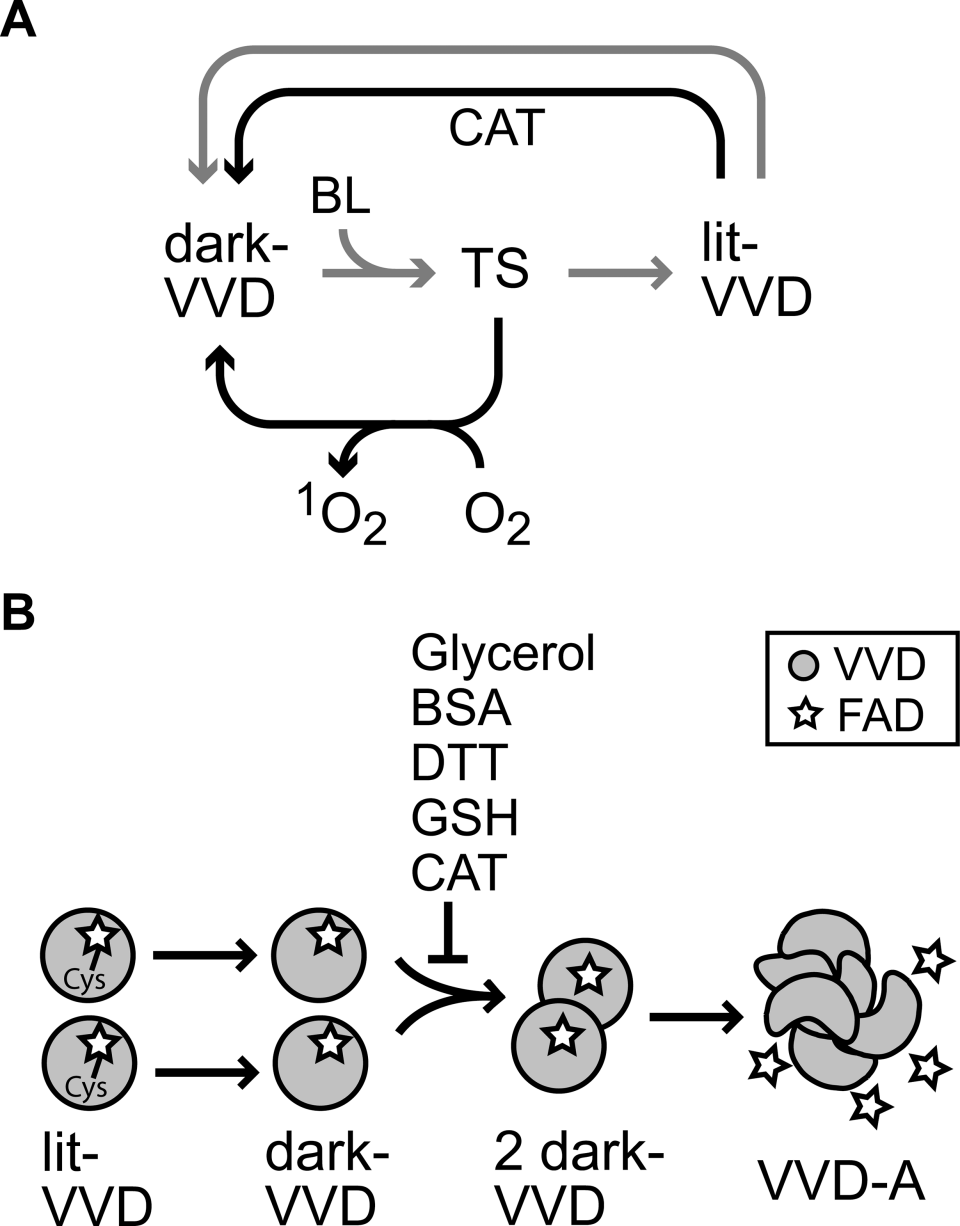
Summary of our findings and proposed models for oxidative damage and aggregation in VVD. (A) Absorption of BL by dark-VVD results in an excited triplet state (TS). In the conventional photocycle (gray arrows) the TS decays to the flavin-cysteine adduct state corresponding to lit-VVD, from where spontaneous adduct decay follows. Alternatively (black arrows), the TS decays to the ground state by energy transfer to O_2_ with the production of ^1^O_2_, which in turn promotes internal chemical damage. This mechanism is expected to occur in other LOV domains. In addition, we discovered that the presence of *A*. *niger* catalase (CAT) accelerates photoadduct decay. (B) The aggregation pathway of self-oxidized VVD is shown. The lit-VVD state decays into dark-VVD, followed by VVD dimerization and formation of aggregates (VVD-A) that lose the flavin cofactor. The aggregation process is under kinetic control (governed by the dynamics of the FAD-Cys adduct) and limited by VVD dimer formation. Glycerol, BSA, DTT, GSH and CAT directly interact with VVD, impeding VVD dimerization and subsequent aggregation.

Our systematic study on the aggregation of VVD reveals alternative outcomes for light exposure of a LOV domain besides the conventional flavin-cysteine photocycle, and reconstructs key elements in the aggregation pathway of a flavin photoreceptor. Although we collected our observations within the context of protein aggregation, specific results arise considerable interest of their own. Among the main findings of our work are two seemingly antagonistic effects induced by light on VVD: self-damaging photosensitizer activity and stability against aggregation. Fig 5 summarizes the main findings of our work.

The production of ^1^O_2_ by LOV domains has been anticipated based on the feasibility of energy transfer from the flavin excited triplet state (TS) to O_2_ [28]. In addition, the quantum efficiency (Φ) for photoadduct formation in LOV proteins upon BL exposure is low (Φ = 0.3–0.6) [9,28,29] even though formation of the preceding TS occurs with Φ = 0.4–0.8 [28], leaving ample possibility for alternative light-driven pathways. Based on our observations, we propose that LOV domains are intrinsically capable of producing ^1^O_2_, by deviating from the conventional photocycle (Fig 5A). Furthermore, the evidence found of internal protein oxidation provides support to the notion that LOV sensors may be subject to chemical damage (and possibly aggregation) as a result of an intrinsic light-sensing activity. Some functional consequences of these effects can be anticipated in native organisms (see below for the case of VVD) as well as in bioengineering applications. Recently, LOV domains have gained notoriety because of their use as reporters for fluorescence imaging and as elements of the optogenetic toolkit [3,30]. However, reports of oxidative damage or aggregation in these proteins are essentially nonexistent and several contributing reasons may be involved. It is possible that the aggregating phenotype could be a specific characteristic of VVD (whose use as a fluorescent reporter has been limited) due to the particular oxidizing agent produced, a lack of stability against oxidation, and the specifics of its relatively long photocycle. Yet, we suggest that self-promoted oxidation and subsequent oligomerization can become major limiting factor towards using LOV domains during extended illumination periods or several photocycles.

To formulate a model for aggregation in VVD, we start with the observation that freshly purified VVD in the dark state presented aggregation (without a lag phase, Fig 3A). Additionally, we show that exposure of VVD to BL increases protein oxidative levels (Fig 2A) and induces production of singlet oxygen (Fig 2C). We therefore conclude that our starting condition is VVD with some level of self-oxidative damage (Fig 2B) caused by VVD photosensitizer activity during protein production. Apart from this aspect, we provide evidence that aggregation proceeds through VVD dimerization, possibly involving exposure of hydrophobic regions as well as formation of disulfide or alternative covalent bonds. The aggregation-prone dimers develop in dark-VVD, and therefore they are most likely different from the light-induced dimers that participate in photoadaptation [31]. Taking into account these considerations, we propose that the aggregation of VVD is due to self-oxidative damage that causes exposure of hydrophobic regions. This loss of stability in turn promotes oligomerization and the development of amorphous, macroscopic aggregates where the flavin cofactor is released.

In addition, a most remarkable effect was discovered: the aggregation of VVD is under kinetic control where the controlling factor is light. The aggregation process of VVD (Fig 5B) thus shows that protein aggregation may depend not only on standard environmental factors (protein concentration, solvents, cosolutes) but also on internal protein dynamics. We hypothesize that the flavin-cysteinyl bond impacts on the network of weak bonds surrounding the FAD cofactor, promoting a compact conformation of VVD that increases protein stability. This conclusion is consistent with a previous report that tested solvent accessibility of the VVD backbone by hydrogen exchange mass spectrometry [32]. A ~36-fold reduction in hydrogen-deuterium exchange times for lit-VVD compared to dark-VVD was found, an effect that could indicate the presence of a compact and not very dynamic VVD core promoted by photoadduct formation. A compact protein would preclude exposure of hydrophobic regions that promote dimer formation towards aggregation and prevent accessibility of denaturants such as SDS. Remarkably, VVD constitutes a rare example where the stability of an oxidized protein is maintained by a single, unstable chemical bond.

Our findings on aggregation, oxidation and kinetic control in VVD present this photoreceptor with possible wider operational mechanisms than previously known. In the context of *N*. *crassa*, VVD has been proposed to participate in diverse physiological responses: regulating photoadaptation [18], integrating temperature into the circadian clock [33], modulating gating and regulating circadian clock resetting [34], and sensing cellular oxidation potential [22]. We speculate that aggregation could therefore be an effective way to regulate VVD function *in vivo* by limiting protein availability and helping maintain an optimum pool of VVD-WCC complexes [35]. This mechanism could be notably at play in photoadaptation. As photoadaptation is difficult to explain based on the sole negative regulation of WCC (the master inducer of photoresponses) by VVD, a recent systems biology approach [36] has proposed that VVD exerts both negative and positive roles in photoadaptation through a “futile cycle” mechanism. In this scheme, the initial sequestration of WCC by VVD both in the lit state (negative regulation) is followed by spontaneous decay of the photoadduct in either VVD or WC-1 thus dissociating the VVD/WCC complex (positive regulation). In view of our results, we hypothesize that: (i) VVD undergoes self-oxidative damage, (ii) even in the presence of damage, the stable lit-VVD state favors effective WCC-VVD formation, (iii) once the VVD dark-state is recovered the interaction N161-V149 (one of six key structural pairs identified in dark-VVD using a computational networks approach [37]) is compromised due to N161 oxidation, consequently decreasing the stability of VVD, and (iv) aggregation of dark-VVD occurs as part of a “terminating futile cycle” that removes the protein due to permanent damage.

The remarkable observation that catalase (from *A*. *niger*) suppressed the aggregation of VVD with concomitant acceleration of VVD photoadduct decay deserves special attention. As high catalase concentrations are needed to observe the effect, we conclude that decomposition of hydrogen peroxide activity is not at play. Instead, we propose direct physical interaction between catalase and VVD, with the effect of lowering the probability for VVD dimerization and subsequent aggregation. Although point mutations, the presence of small bases (such as imidazole) and pH have been shown to tune adduct photodecay rates in BL receptors [38,39], ours constitutes, to our knowledge, the first report of a protein altering the photocycle dynamics of a LOV photoreceptor. The modification of VVD adduct lifetime by catalase suggests the tantalizing possibility of direct redox interaction between these two proteins. Further work will clarify the nature of this interaction and its importance for physiological processes where VVD or catalase are involved.

Finally, the stability of VVD kinetically controlled by light offers new insight into the role of the flavin-cysteine adduct in fungal photoresponses. Recently it was found that LOV variants without the reactive cysteine show evidence of functional activity by means of flavin photoreduction, suggesting that formation of the photoadduct could be spared during light signaling [40]. What is then the role of the photoadduct in VVD? We propose that as VVD is under significant risk of self-induced oxidation due to intrinsic photosensitizer activity of the FAD cofactor, the flavin-cysteinyl bond plays a protective role by establishing a kinetic barrier that retains a compact protein conformation and activity despite chemical damage. This feature may be shared by other proteins harboring LOV domains, where due to the highly variable reactivity of oxygen with flavoproteins production of self-damaging ROS is expected to present [27].

## Supporting information

S1 File (PDF)
