# Supplementary material for: Light induces oxidative damage and protein stability in the fungal photoreceptor Vivid
Source: PLoS One. 2018 Jul 20;13(7):e0201028. doi: 10.1371/journal.pone.0201028 (PMC6054393; doi:10.1371/journal.pone.0201028)
Supplement: S1 File — (PDF) [file pone.0201028.s001.pdf]

Supporting Information for

Light induces oxidative damage and protein stability against aggregation in the fungal photoreceptor Vivid

Authors: C.N Hernández Candia, S. Casas-Flores, and B. Gutiérrez-Medina*

*To whom correspondence should be addressed. E-mail: bgutierrez@ipicyt.edu.mx

List of Contents:

- SI Text
- Figure A. The flavin cofactor is released during aggregation of VVD.
- Figure B. Standard recombinant protein troubleshooting does not prevent the aggregation of VVD.
- Figure C. Mascot (MS/MS) search results from analysis of an aggregated VVD sample.
- Figure D. Dose-dependent effect of ROS-scavengers and antioxidants.
- Figure E. Absorbance spectra of VVD samples initially prepared in the lit or dark states.
- Figure F. Absorbance spectra of VVD samples subject to re-illumination.
- Figure G. The aggregation rate of VVD is dependent on protein concentration.
- Figure H. Glycerol and BSA effects over VVD aggregation kinetics.
- Figure I. Incubation with DTT limits formation of VVD oligomers during aggregation.
- Figure J. The aggregation of VVD does not depend on its conventional conformational change.

SI Text

Standard troubleshooting to avoid the aggregation of VVD

The most common recombinant-protein aggregation troubleshooting tests were conducted to avoid the observed aggregation of 6×His-VVD36 at room temperature (Figure B).

First, different buffer conditions were explored: 1) We removed imidazole from freshly purified protein by passing samples through a PD10 column (Figure B). 2) The protein standard buffer was replaced for a phosphate buffer using the PD10 column (Figure B). 3) Different concentrations of NaCl in the standard buffer were tested (data not shown). None of the tested buffer conditions avoided the aggregation of VVD. Second, to discard a possible metal-catalyzed protein oxidation (1) due to the nickel involved in protein purification using a Ni-NTA resin, following Ni-NTA purification the protein was passed through a chelex 100 resin (SIGMA, C7901). This procedure did not avoid protein aggregation (Figure B).

Second, the N-terminal 6×His tag was removed using the Thrombin CleanCleave Kit (SIGMA, RECOMT). SDS-PAGE was used to confirm that the 6×His tag was effectively cleaved (Figure B). The absorbance spectra of the free-tag protein were monitored, and the corresponding absorbance kinetics at 550 nm shows that aggregation is still present (Figure B).

Finally, to fully discard any effect due to the 6×His tag or the 6×His purification method, a double-tagged 36VVD version with a 6×His tag at the N-terminus and a Strep-tag at the C-terminus (plasmid purchased to DNA2.0) was purified using a Strep-Tactin Superflow Plus resin (QIAGEN, G00016). A western blot against 6×His of the double-tagged VVD protein was performed to confirm the identity of the protein (Figure B). The protein purified using Strep-Tag was illuminated for 5 min with blue light and absorption spectra were acquired, showing the shift in the absorbance at 550 nm that is indicative of protein aggregation (Figure B).

SI References

1. Stadtman, E R (1990) Metal ion-catalyzed oxidation of proteins: bio-chemical mechanism and biological consequences. Free Radic. Biol. Med. 9(4):315–325.

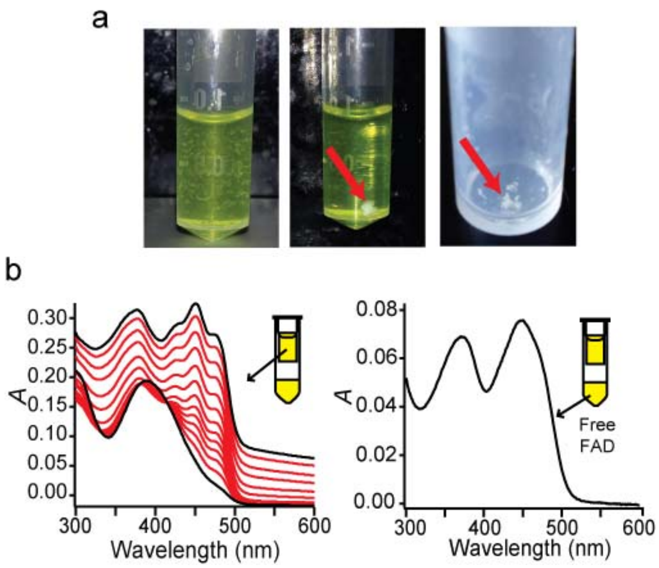

Figure A. The flavin cofactor is released during aggregation of VVD. (a) After incubation of 24 h at 25 °C a VVD sample became visibly turbid. A whitish pellet is formed after spin down of the turbid sample (red arrows), suggesting loss of the flavin cofactor. (b) The supernatant of the spin down aggregated sample was passed through an Amicon centrifugal filter and samples from the upper (retained) and lower (through) part of the filter were recovered and their respective absorption spectra were recorded.

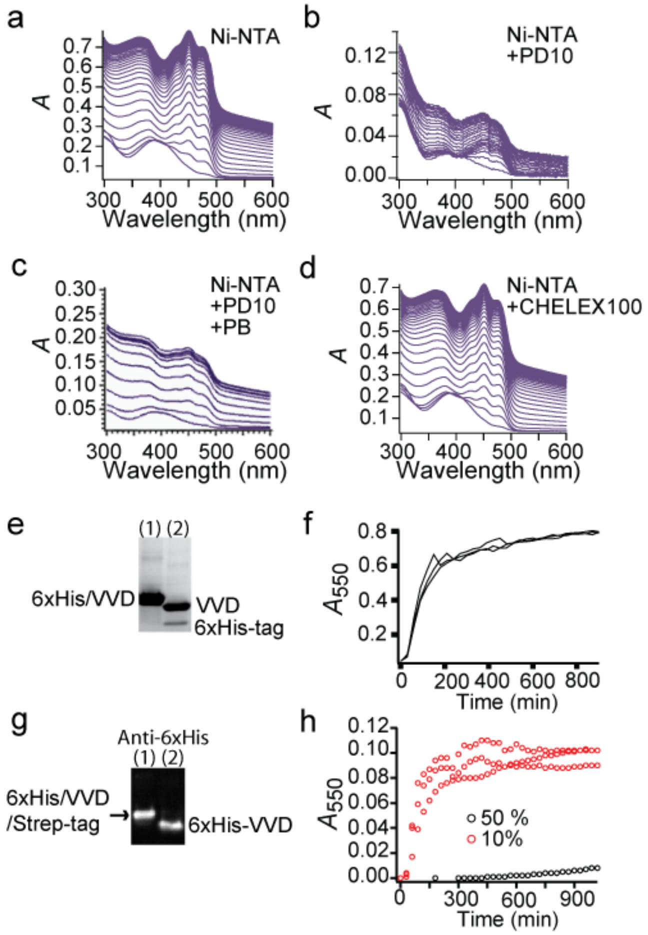

Figure B. Standard recombinant protein troubleshooting does not prevent the aggregation of VVD. (a-d) Absorbance spectra of 6×His-VVD36 (at 10% (v/v) glycerol, room temperature) after: purification using a Ni-NTA column (a), purification using a Ni-NTA column and desalting using a PD10 column (b), purification using a Ni-NTA column and buffer exchange to potassium phosphate buffer (PB, 50 mM, pH 7.0) using a PD10 column (c), and purification using a Ni-NTA column and residual Ni removal using a CHELEX column (d). (e) SDS-PAGE of a 6×His-VVD36 protein after (line 1) and before (line 2) thrombin digestion that removed the 6×His-tag. (f) Absorption kinetics at 550 nm of 6×His-free VVD. (g) Western blot against the 6×His of 6×His-VVD-Streptag and 6×His-VVD. (h) Absorption kinetics at 550 nm of the double-tag VVD protein purified by its Streptag. Percentages indicate amount (v/v) of glycerol present in samples.

Protein sequence coverage: 36%

Matched peptides shown in light gray.

```
  1 MSHTVNSSTM NPWEVEAYQQ YHYDPRTAPT ANPLFFHTLY APGGYDIMGY
 51 LIQIMNRPNP QVELGPVDTS CALILCDLKQ KDTPIVYASE AFLYMTGYSN
101 AEVLGRNCRF LQSPDGMVKP KSTRKYVDSN TINTMRKAID RNAEVQVEVV
151 NFKKNGQRFV NFLTMPVRD ETGEYRYSMG FQCETE
```

Unformatted sequence string: 186 residues (for pasting into others application)

Sort by ◉ residue number ○ increasing mass ○ decreasing mass
Show ◉ matched peptides only ○ predicted peptides also

Query	Start – End	Observed	Mr(expt)	Mr(calc)	ppm	M	Score	Expect	Rank	U	Peptide
251	82 – 106	923.1083	2766.3031	2766.3211	-6.50	0	63	8.5e-05	1	U	K.DTPIVYASEAFLYMTGYSNAEVLGR.N
254	82 – 106	923.1095	2766.3067	2766.3211	-5.20	0	38	0.046	1	U	K.DTPIVYASEAFLYMTGYSNAEVLGR.N
279	82 – 106	928.4411	2782.3015	2782.3160	-5.21	0	27	0.031	1	U	K.DTPIVYASEAFLYMTGYSNAEVLGR.N + Oxidation (M)
280	82 – 106	1392.1587	2782.3028	2782.3160	-4.72	0	97	5.2e-05	1	U	K.DTPIVYASEAFLYMTGYSNAEVLGR.N + Oxidation (F)
281	82 – 106	1392.1592	2782.3038	2782.3160	-4.36	0	99	7e-08	1	U	K.DTPIVYASEAFLYMTGYSNAEVLGR.N + Oxidation (Y)
282	82 – 106	928.4427	2782.3063	2782.3160	-3.49	0	63	0.0012	1	U	K.DTPIVYASEAFLYMTGYSNAEVLGR.N + Oxidation (D)
285	82 – 106	1392.1617	2782.3088	2782.3160	-2.56	0	109	1.4e-08	1	U	K.DTPIVYASEAFLYMTGYSNAEVLGR.N + Oxidation (F)
286	82 – 106	1392.1619	2782.3092	2782.3160	-2.42	0	116	7.4e-09	1	U	K.DTPIVYASEAFLYMTGYSNAEVLGR.N + Oxidation (Y)
289	82 – 106	1392.1650	2782.3154	2782.3160	-0.19	0	95	7.3e-05	1	U	K.DTPIVYASEAFLYMTGYSNAEVLGR.N + Oxidation (Y)
290	82 – 106	1392.1653	2782.3160	2782.3160	0.025	0	100	6.4e-08	1	U	K.DTPIVYASEAFLYMTGYSNAEVLGR.N + Oxidation (Y)
291	82 – 106	1392.1660	2782.3174	2782.3160	0.53	0	110	8.4e-09	1	U	K.DTPIVYASEAFLYMTGYSNAEVLGR.N + Oxidation (F)
292	82 – 106	1392.1664	2782.3182	2782.3160	0.82	0	93	0.00011	1	U	K.DTPIVYASEAFLYMTGYSNAEVLGR.N + Oxidation (F)
131	125 – 136	481.2350	1440.6832	1440.7031	-13.8	1	57	0.045	1	U	R.KYVDSNTINTMR.K
50	126 – 136	657.3142	1312.6138	1312.6081	4.35	0	89	1.5e-06	1	U	K.YVDSNTINTMR.K
59	126 – 136	665.3035	1328.5924	1328.6031	-7.99	0	53	0.042	1	U	K.YVDSNTINTMR.K + Oxidation (M)
87	142 – 153	688.3607	1374.7068	1374.7143	-5.44	0	38	0.024	1		R.NAEVQVEVVNFK.K
144	142 – 154	501.9344	1502.7814	1502.8093	-18.6	1	87	1.7e-06	1		R.NAEVQVEVVNFKK.N
146	142 – 154	752.4054	1502.7962	1502.8093	-8.67	1	66	0.00029	1		R.NAEVQVEVVNFKK.N
193	159 – 176	729.6955	2186.0647	2186.0830	-8.39	1	77	3.9e-06	1	U	R.FVNFLTMPVRDETGEYR.Y
194	159 – 176	729.6965	2186.0677	2186.0830	-7.02	1	81	6.9e-06	1	U	R.FVNFLTMPVRDETGEYR.Y
204	159 – 176	735.0265	2202.0577	2202.0779	-9.20	1	79	2.2e-05	1	U	R.FVNFLTMPVRDETGEYR.Y + Oxidation (F)
205	159 – 176	735.0289	2202.0649	2202.0779	-5.94	1	72	0.00067	1	U	R.FVNFLTMPVRDETGEYR.Y + Oxidation (N)
211	159 – 176	740.3530	2218.0372	2218.0729	-16.1	1	78	0.0022	1	U	R.FVNFLTMIPVRDETGEYR.Y + Oxidation (F); Oxidation (M)
212	159 – 176	740.3607	2218.0603	2218.0729	-5.68	1	82	0.0011	1	U	R.FVNFLTMIPVRDETGEYR.Y + Oxidation (F); Oxidation (M)

Figure C. Mascot (MS/MS) search results from analysis of an aggregated VVD sample.

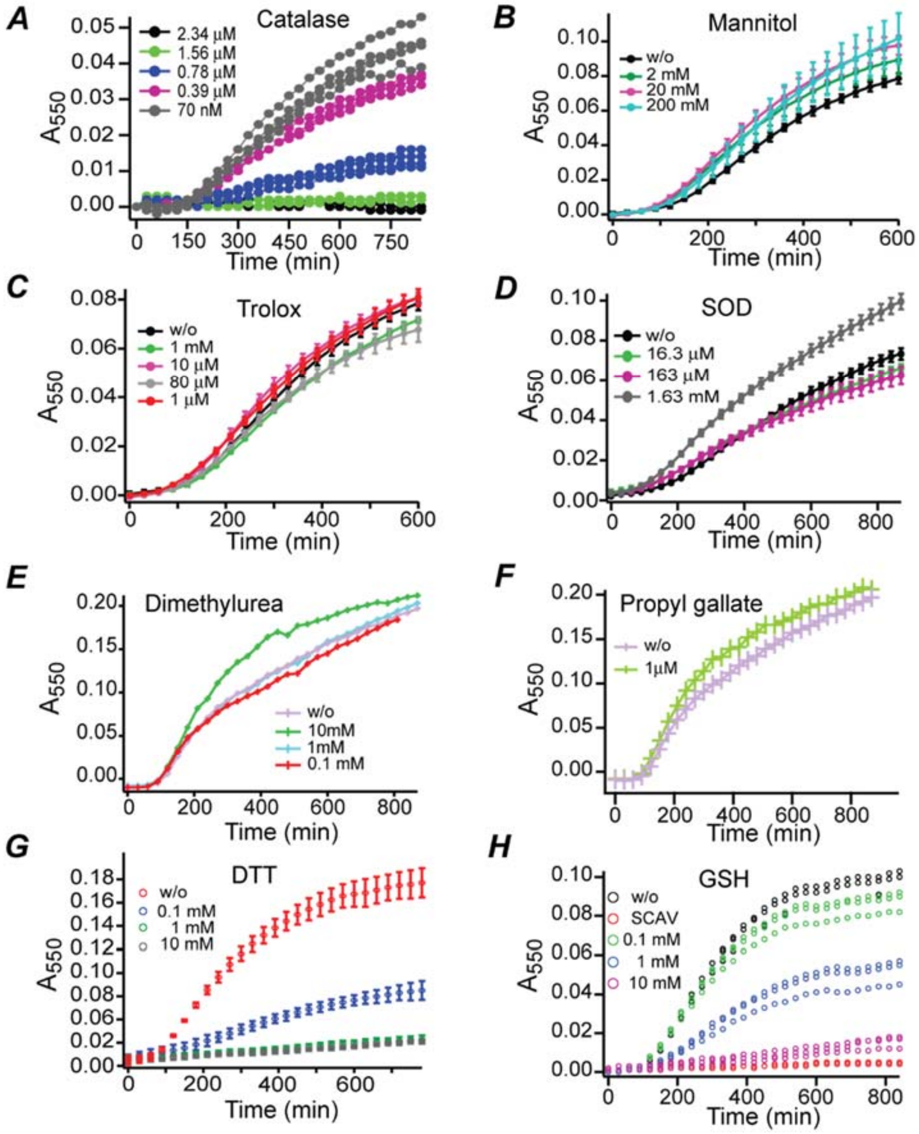

Figure D. Dose-dependent effect of ROS-scavengers and antioxidants. All samples were illuminated for 5 min with blue light and the absorbance at 500 nm was recorded every 30 min (N=3, mean ± SD). The scavengers tested were: catalase (CAT) (A), mannitol (B), trolox (C), superoxide dismutase (SOD) (D), Dimethylurea (E), Propyl gallate (F), DTT (G) and GSH (H). For the SOD condition 156 nM of catalase was added to eliminate hydrogen peroxide produced by superoxide dismutase. Kinetics corresponding to VVD protein samples in standard buffer conditions without the respective molecule under test are also shown for reference (w/o).

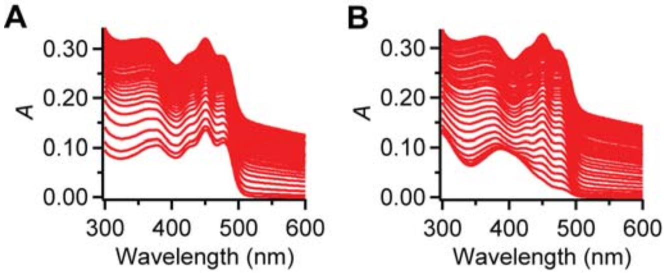

Figure E. Absorbance spectra of VVD samples initially prepared in the dark (A) or lit (B) states. Measurements were performed every 890 s.

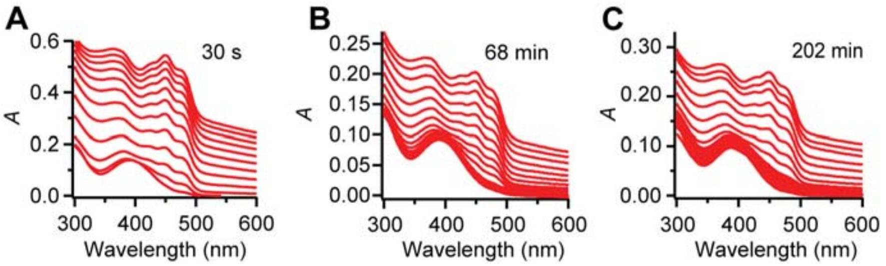

Figure F. Absorbance spectra of VVD samples subject to re-illumination. All samples were illuminated initially during 30 s. (A) A reference sample was left to aggregate after exposure to only one light pulse. Each spectrum was acquired every 1608 s. (B and C) Samples were re-illuminated every 5 min during 68 min (B) and 202 min (C). After the re-illumination period samples were left in dark conditions and spectra were recorded every 12 min.

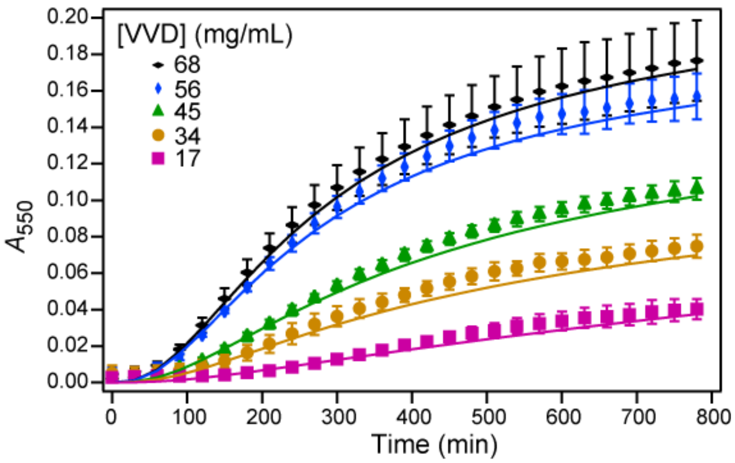

Figure G. The aggregation rate of VVD is dependent on protein concentration. VVD samples prepared at different initial protein concentrations in standard buffer (150 mM NaCl, 50 mM HEPES, 10% glycerol, 20 mM imidazole, pH 8) were exposed to a blue-light pulse and their corresponding absorption spectra recorded every 30 min (symbols). Data is consistent with our proposed model that considers photoadduct decay as the limiting step for VVD aggregation (fits to Equation 2 in the main text, solid lines). Fitting values: $\tau = 3.4 \times 10^3$ s for all records and k_1 = 2.4, 2.3, 1.7, 0.9, and 0.2 M^{-1} s^{-1} for [VVD] = 68, 56, 45, 34, and 17 mg mL^{-1}, respectively. Data: mean, error bars: SD; $n = 3$

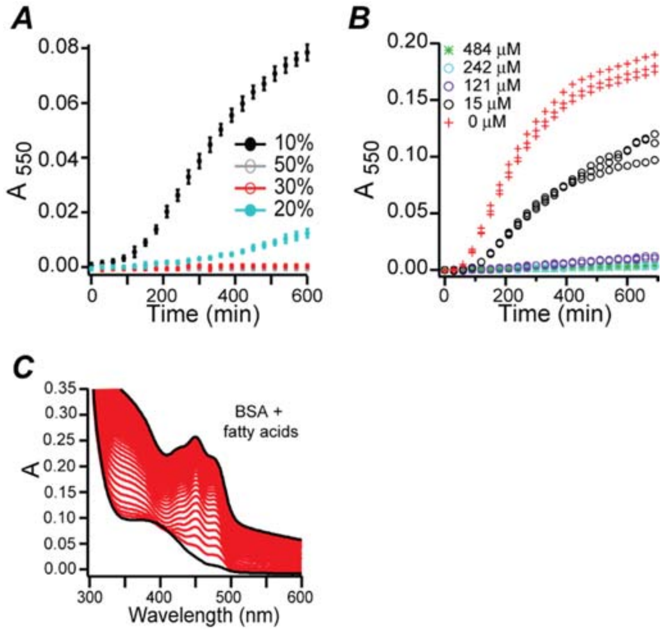

Figure H. Glycerol and BSA effects over VVD aggregation kinetics. VVD aggregation kinetics, measured as absorbance at 550 nm, for protein (A) resuspended in different concentrations of glycerol (mean ± SD, N=3), and (B) incubated with different amounts of the chaperon protein BSA (N=3). Increased glycerol or BSA concentrations decrease aggregation rates with consequent final absorbance shifts after overnight incubation. (C) The effect of BSA on VVD aggregation is abated by fatty acids. VVD samples were supplemented with linoleic acid-oleic acid-albumin at a final concentration of 242 μM BSA and 484 μM each fatty acid.

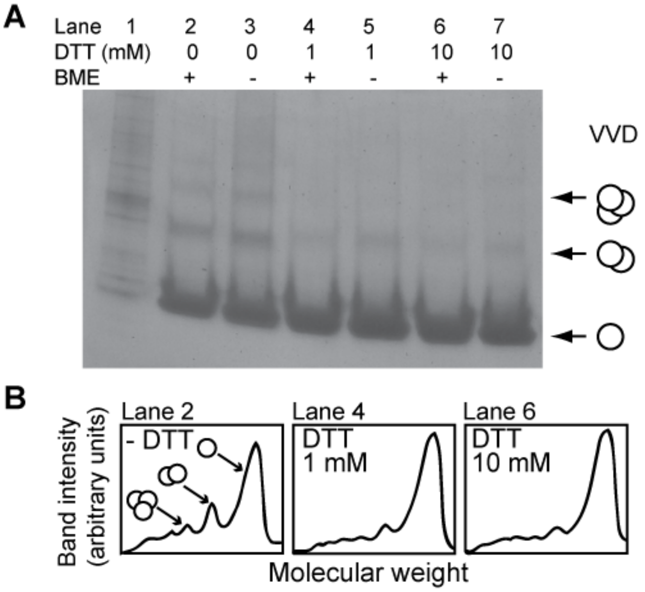

Figure I. Incubation with DTT limits formation of VVD oligomers during aggregation. (A) VVD samples were left to aggregate in the absence or presence of the well-known disulfide-bond disruptor DTT. Results of SDS-PAGE of samples are shown. Beta mercaptoethanol (BME) added after protein aggregation shows little effect, indicating that VVD oligomers are stable after formation. In the absence of DTT (lanes 2 and 3) VVD oligomers are easily identified. As the reducing agent DTT is added (lanes 4-7) the presence of VVD oligomers is much lowered. (B) Band intensity profiles corresponding to lanes 2, 4 and 6. Lane 1 corresponds to protein ladder.

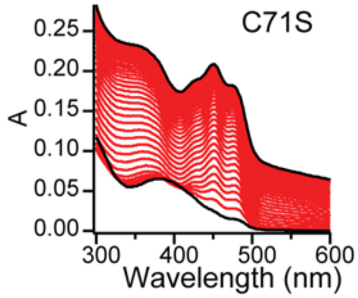

Figure J. The aggregation of VVD does not depend on its conventional conformational change. Purified VVD C71S (that does not perform the well-known light-driven conformational change) was subjected to one initial blue-light pulse. Subsequent absorption spectra taken every 30 min also present increase in turbidity, indicative of aggregation.
